# Preoperative Anxiolysis and Treatment Expectation (PATE Trial): open-label placebo treatment to reduce preoperative anxiety in female patients undergoing gynecological laparoscopic surgery – study protocol for a bicentric, prospective, randomized-controlled trial

**DOI:** 10.3389/fpsyt.2024.1396562

**Published:** 2024-07-09

**Authors:** Johannes Wessels, Regine Klinger, Sven Benson, Thorsten Brenner, Sigrid Elsenbruch, Jana L. Aulenkamp

**Affiliations:** ^1^ Department of Anesthesiology, University Medical Center Hamburg Eppendorf, Hamburg, Germany; ^2^ Institute for Medical Education, Center for Translational Neuro- and Behavioral Sciences (C-TNBS), University Hospital Essen, University of Duisburg-Essen, Essen, Germany; ^3^ Department of Anesthesiology and Intensive Care Medicine, University Hospital Essen, University Duisburg-Essen, Essen, Germany; ^4^ Department of Neurology, Center for Translational Neuro- and Behavioral Sciences (C-TNBS), University Hospital Essen, University Duisburg-Essen, Essen, Germany; ^5^ Department of Medical Psychology and Medical Sociology, Ruhr University Bochum, Bochum, Germany

**Keywords:** placebo, treatment expectation, postoperative pain, anxiolysis, visceral pain, somatic pain, fear, surgery

## Abstract

One of the most common concerns of patients undergoing surgery is preoperative anxiety, with a prevalence of up to 48%. The effects of preoperative anxiety continue beyond the preoperative period and are associated with more severe postoperative pain and poorer treatment outcomes. Treatment options for preoperative anxiety are often limited as sedatives cause side effects and their efficacy remains controversial. Placebo research has shown that optimization of positive treatment expectations, as can be achieved through placebo administration and education, has clinically relevant effects on preoperative anxiety, pain and treatment outcomes. As the administration of masked placebos raises ethical questions, clinical studies have increasingly focused on the use of open, non-deceptive placebo administration (open-label placebo, OLP). The use of OLPs to reduce preoperative anxiety and modify clinically relevant postoperative outcomes has not yet been investigated. This bicentric, prospective, randomized-controlled clinical trial (PATE Trial; German Registry for Clinical Studies DRKS00033221), an associated project of the Collaborative Research Center (CRC) 289 “Treatment Expectation”, aims to alleviate preoperative anxiety by optimizing positive treatment expectations facilitated by OLP. Furthermore, this study examines a potential enhancement of these effects through aspects of observational learning, operationalized by a positive expectation-enhancing video. In addition, patient’s perspective on the self-efficacy and appropriateness of OLPs prior to surgery will be assessed. To achieve these objectives, female patients will be randomized into three groups before undergoing gynecological laparoscopic surgery. One group receives the OLP with a positive rationale conveyed by a study physician. A second group receives the same intervention, OLP administration and rationale provided by a physician, and additionally watches a video on OLP presenting a satisfied patient. A third group receives standard treatment as usual (TAU). Outcome measures will be effects on preoperative anxiety and postoperative experience, particularly visceral and somatic postoperative pain. As the non-deceptive administration of placebos; when indicated; may yield positive outcomes without side effects, and as current treatment of preoperative anxiety is limited, evidence from clinical placebo research has the potential to improve outcomes and patient experience in the surgical setting.

## 1 Introduction

Preoperative anxiety is a very common burden for patients before surgery, with a prevalence of up to 48% (1–3). Up to 318 million surgeries were performed worldwide in 2012, with increasing global volume (4) potentially leading to over 160 million patients being affected by anxiety in the perioperative setting. Preoperative anxiety and the associated psychological stress burden manifests in increased anesthesia requirements (5) and subsequent consequences such as haemodynamic instability, impaired postoperative cognitive and physical recovery (6), prolonged recovery time and prolonged hospital stay (7). In addition, the effects persists after surgery, leading to increased postoperative pain (8, 9), which is a crucial risk factor for persistent postoperative pain (10). Moreover, higher preoperative anxiety is associated with increased opioid postoperative consumption (11) and a lower quality of life after surgery (8, 9). Also, preoperative anxiety appears to be predictor of the occurrence and level of postoperative anxiety (12). Consequently, perioperative anxiety incorporates the anxiety that occurs both before and after a surgical procedure.

Female gender and gynecological surgery are associated with more prevalent and severe preoperative anxiety (13), making this patient group of particular interest. Reducing preoperative anxiety can improve surgical outcomes, shorten hospital stay and reduce negative impact on quality of life, calling for interventions aimed at improving preoperative anxiety. Current treatment regimens are mostly based on pharmacological interventions with unfavorable side effects (14), resulting in a decrease in prescriptions (3, 6). Therefore, anesthesiologists have a crucial responsibility in guiding patients through the process between preoperative anxiety, the anesthetic as well as surgical procedure, limited treatment options and uncertain treatment success.

Placebo research already provides convincing evidence that psychological preparation for surgery has a positive effect on postoperative pain and the length of hospital stay (15). Experimental and clinical studies reveal clinically relevant treatment effects on anxiety or pain elicited by positive treatment expectations, for example by placebo pills or placebo interventions (2, 16–19). Negative expectation effects modulated by anxiety, demonstrably shape treatment outcomes, including patient-reported pain and treatment outcome (20–22). In addition, abdominal surgery can cause visceral pain, deep in the abdomen, and superficial somatic pain (23), and especially visceral pain is substantially amplified by negative emotions and cognitions, such as fear (24) or stress (25).

Placebo treatments without deception, known as open-label placebo (“OLP”), meaning patients know and agree that they are receiving a placebo, can also produce positive treatment effects (26–29). A meta-analysis of OLPs in patients with back pain, cancer-related fatigue, attention-deficit hyperactivity disorder, allergic rhinitis, major depression, irritable bowel syndrome, and menopausal hot flushes found an overall significant, moderate-sized effect of OLPs in these eleven RCTs. Such effects also include reducing test anxiety and improving self-management skills like coping, self-efficacy, introspection, hope, and self verbalization (30). Beyond that, taking OLPs alleviated pain after their administration and reduced the need for analgesics after surgery (31, 32). Positive and negative expectancy effects on treatment outcome also include placebo hypoalgesia, which refers to a reduction in perceived pain due to the psychological effects of receiving a placebo believed to be an analgesic (33), and have great potential for use in clinical settings.

Several mechanisms are currently recognized as underlying expectancy effects: Verbal information or instruction provided by healthcare professionals (cognitive model), classical conditioning (associative learning) and social learning (observational learning) (16), which generate the expectation-mediated placebo effect (34). The specific mechanisms underlying the effects of OLPs are not yet fully understood (35). However, there is evidence that these mechanisms are similar to those of deceptive placebo. When OLPs are used as an adjunctive treatment, the effect may rely on placebo responses induced by classical conditioning (36). Also, the effects of OLPs are often elicited by verbal suggestion, highlighting the important role of doctor–patient interaction and cognitive processing in the efficacy of OLP. In contrast to deceptive placebos, OLP treatments do not require patients to be blinded and promote awareness of the treatment, thereby enhancing autonomy and potentially activating the body’s self-regulatory mechanisms through conditioned responses (37). Furthermore, it is hypothesized that the success of OLP treatments is highly dependent on the active participation of the patient, whereby the recognition of the placebo (“the pill is without medication but not without effect”) may require cognitive flexibility to effectively reconcile the paradoxical information (38). Another theoretical framework for OLPs is the “Bayesian brain” model, which suggests that OLP helps to resolve cognitive dissonance by adjusting expectations based on new evidence (39). Given the limited knowledge of the mechanisms, the patients’ perspective on this novel treatment is of particular interest for the future understanding.

Treatment expectations can also arise from previous treatment experiences, as well as information from the media or peers. Our research group has investigated the modulation of expectations by verbal instructions and conditioning in experimental human and patient studies (17, 21, 32, 40, 41), but mainly experimental studies on observational learning in placebo or nocebo treatment have been conducted (33). However, we have also begun to investigate observational learning in patient populations. We were able to provide evidence that clinically meaningful effects can be achieved by patients observing placebo effects in an actor patient and changing expectations in chronic pain patients (42). Therefore, the encouraging benefits of observational learning derived from preclinical research provide an incentive to further investigate the role of observational learning as an important objective of our clinical trial concept and design in this patient population.

Reducing anxiety in the preoperative period is of utmost importance in order to reduce the risk of perioperative complications and ultimately postoperative pain after surgery. Treatment expectations have not yet been systematically studied, although a refined assessment of positive and negative expectations may complement other psychosocial risk factors in determining individual risk for adverse health outcomes, e.g. based on concern of adverse events or low self-efficacy expectations regarding the ability to cope with preoperative anxiety and postoperative pain. More importantly, it appears that expectations can be optimized. The aim of this project is to increase knowledge regarding how treatment expectations and related mechanisms can be specifically improved in a clinical context in order to improve treatment outcomes.

Therefore, we designed a bicentric randomized-controlled clinical trial dedicated to elucidate the optimization of treatment expectations using OLPs to reduce preoperative anxiety and to improve postoperative outcomes in gynecological patients. Patients will be randomized into three groups prior to laparoscopic gynecological surgery. The design allows for testing the effects of the OLP versus standard care. In addition, we will test whether the effects of OLP can be enhanced by social observational learning, e.g. via a video presenting information about OLP from a satisfied patient. The primary outcome will be preoperative anxiety. Secondary outcomes will include postoperative visceral and somatic pain, sedation requirements and analgesic consumption. Additionally, the perspectives of patients and healthcare providers on the use of OLPs will be evaluated.

Specific objectives of this study:

Objective 1: To compare preoperative anxiety after standard treatment (TAU-Group) against modulation of treatment expectations using OLP (OLP-Group), and to determine whether the effects after modulation of treatment expectation using OLPs can be enhanced by a video with aspects of verbal suggestion and social learning (OLP+V-Group).
Objective 2: To compare dynamic changes in anxiety and treatment expectation over time in the three groups.
Objective 3: Comparison of group differences in the pre- and postoperative course, in particular patient-reported visceral and somatic postoperative pain intensity, sedative, anesthesia and analgesic requirements, duration of anesthesia induction and hospital stay.
Objective 4: To explore the patients and health care providers perspective regarding the use of OLPs to reduce anxiety before surgery.

## 2 Methods

### 2.1 Setting

This bicentric study is conducted at the University Hospital Essen, University Duisburg-Essen, Germany and the University Hospital Hamburg-Eppendorf, University of Hamburg, Germany as affiliated project of the Collaborative Research Center (CRC) 289 “Treatment Expectation”, funded by the German Research Foundation (Deutsche Forschungsgemeinschaft, DFG).

The overall goal of the CRC is to elucidate mechanisms and clinical implications of treatment expectations (https://treatment-expectation.de/en/). The present study is accomplished as cooperation as part of the subproject A04 (PI: author S.E.) and of the subproject A13 (PI: author R.K.). Ethical approval for this study was obtained in coordinated process of the ethics committees at University Hospital Essen (23–11529-KOBO) and Hamburg (2023–200843-BO-kV-bet). This clinical study has been registered in the German Registry for Clinical Studies (DRKS: DRKS00033221). This protocol (Version 1, 10.06.2024) followed the recommendations for the content of a clinical trial protocol; Standard Protocol Items: Recommendations for Interventional Trials (SPIRIT) and the template for intervention description and replication (TIDieR) checklist and guide.

All patients are required to give informed consent and a physical examination is performed before study enrollment.

### 2.2 Patients and recruitment

The recruitment goal is to include a total of N=144 female patients with planned gynecology laparoscopy. To this end, women between 18 and 70 years of age and an ASA Classification from I–III are recruited before the surgery in the department of Anesthesiology and Intensive Care Medicine at the University Hospital Essen, Germany and in the department of Anesthesiology at the University Medical Center Hamburg Eppendorf, Hamburg, Germany.

As part of the standardized recruitment process, we review the operating schedules of the hospitals in advance and consult with the respective gynaecology departments regarding potential candidates. Patients are offered participation in the study during the premedication visit. Exclusion criteria include any acute major psychiatric illnesses (severe episodes of anxiety, affective disorders, psychosis, or acute exacerbations of substance abuse), a suspected highly malignant tumor requiring surgery to remove one or more than one organ, benzodiazepines in long-term medication defined as regular daily intake according to the medication plan at least one month, previous serious anesthesia-related complications (e.g. difficult airway with anesthesia problem card), expected difficult airway leading to fiberoptic awake intubation as a particularly anxiety-inducing procedure or rare hereditary galactose intolerance, lactase deficiency or glucose-galactose malabsorption due to the ingredients of the placebo pill. In addition, an insufficient proficiency in the German language and in retrospect severe peri- and postoperative complications (e.g. life-threatening condition with unplanned admission to the intensive care unit or death) leading to exclusion. History of drug use and abuse is taken during the premedication visit and taken into account for our study.

### 2.3 Design and groups

**Figure 1:**
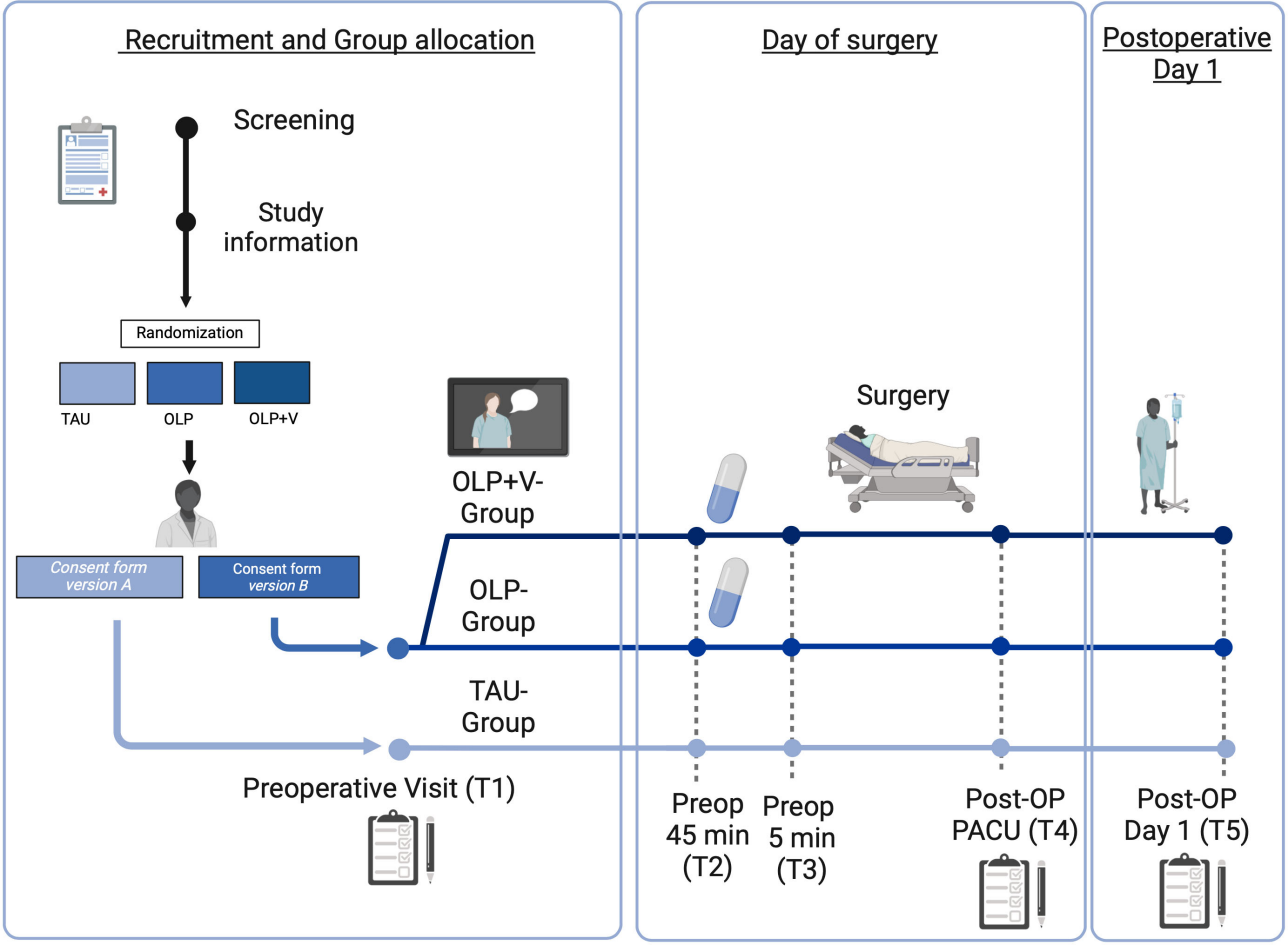
Graphical overview of the study schedule. OLP, open-label placebo; OP, operation; preop, preoperative; PACU, post anesthesia care unit; T, time point. OLP+V, open-label placebo + video.

The study design of this bicentric, prospective, randomized, controlled, interventional trial is visualized in **Figure 1**, and procedures are described in detail in subsequent sections. Female patients are randomized into three groups before undergoing gynecological laparoscopic surgery. Randomization is accomplished prior to the personal interview with the study physician. To randomize the patients, we use block randomization with random distribution to ensure a balance in sample size across groups over time and the ability to perform interim analysis. Stratification is performed within the Hamburg and Essen clinic locations.

Study group 1 receives the OLP with a positive rationale conveyed by a study physician (OLP-Group; for details see 3.4.1). We will use white pills (P-Tabletten weiß 10 mm Lichtenstein, Zentiva Pharma GmbH, Frankfurt, Germany) as Open-Label Placebo pills. To test effects of social observational learning aspects, study group 2 receives the identical treatment as group 1, and additionally watches a video showing a patient who receives OLP treatment and reports to be highly satisfied with its effects on preoperative anxiety (OLP+V-Group; for details see 3.4.1.1). A third group undergoes the standard care (“treatment as usual”, TAU-Group). Of note, patients are not always given medication to reduce anxiety in standard care. However, it is standard procedure in both clinics for patients to be given a benzodiazepine on request or if they report severe concerns or anxiety. Side effects and contraindications will be considered and discussed with the patients. If a benzodiazepine is prescribed, its standard dose is 7.5 mg Midazolam p.o., only if the patient weighs less than 50kg a reduction in dose will be accomplished. In the OLP-Group, patients receive the OLP first and the anxiolytic only if they still require it afterwards, which will be documented and considered in analysis. The impact of anxiolytic medication on the results of the study will be specifically analyzed to determine whether its inclusion or exclusion from the dataset affects the results, ensuring a careful assessment of its impact on the overall effectiveness of the OLP.

### 2.4 Study procedures

Patients undergoing elective gynecological laparoscopy at the University Hospitals of Hamburg and Essen are offered to participate in the study. After consultation with an anesthesiologist who is not involved in the study, patients are informed about the study, clinically examined and informed consent is obtained by a study physician, an experienced anesthesiologist. In all groups, information about the study is provided by the study physicians in the premedication outpatient clinics individually, which is typically one to five days before the surgery at both study sites.

Patients are assessed at 5 predefined time points. After informed consent, patients complete a comprehensive questionnaire battery (T1). This package includes information on demographics, state and trait anxiety, pain level over the past 4 weeks, treatment expectations regarding to the upcoming surgery and, if applicable, expectations concerning OLP (for details see 3.5). Patients will complete a short questionnaire at the beginning and at the end of the consultation with the study physician, about their current anxiety and treatment expectations in order to assess the impact of the study physician’s suggestions, including the rationale for OLP. Additionally, the patients rate the warmth and competence of the study physician using a standardized questionnaire. Patients in the OLP+V-Group watch the video after the consultation with the study physician and are also subsequently asked to answer questions about their perception of the video.

At the time the patients are called to the operating room, approximately 45 minutes before the start of surgery (T2), patients randomized to the OLP groups receive OLPs from the nursing staff. In addition, the patients evaluate their preoperative anxiety. Just before the induction of anesthesia in the preoperative phase (T3), preoperative anxiety is assessed again. In addition, the anesthetist subjectively assesses the patients’ level of calmness or anxiety. After surgery in the recovery room, patients are asked about their current level of anxiety and about their current pain level for visceral and somatic pain modalities (T4).

On the first postoperative day (T5), patients report if they took the OLPs, their current anxiety and retrospectively their anxiety before surgery. They assess mobility and their general condition. Current pain, visceral and somatic pain modalities as well as discomfort are also recorded. In addition, the warmth and competence of the anesthesiologist performing anesthesia in interacting with the patient and the patient’s perspective on the acceptability of OLPs is recorded.

The patients in the TAU-Group also receive the questionnaires at the same time points, and otherwise no differences from standard care are made. Patients can withdraw their participation in the study at any time.

#### 2.4.1 Treatment instruction

Depending on the intervention group, participants receive appropriate written and verbal treatment information, and sign distinct versions of consent forms. The TAU-Group receives *consent form version A*, explaining the participation in a questionnaire study on preoperative anxiety. The two OLP groups receive *consent form version B*, explaining the use of an OLP to support well-being and to reduce anxiety before surgery. In version B, the possibility is mentioned that the patients will additionally be shown an information video.

All treatment-related information is provided by the study physicians (authors J.L.A. and J.W., respectively) according to a highly standardized protocol. For OLP instruction groups, the rationale protocol is based on a previously used approach of positive framing with the aim of optimizing placebo response (26). In addition, we considered studies of patient perspective on OLPs (43, 44) and obtained feedback from non-participating patients. Established communication strategies were adopted from previous reports (18), including warm and empathic interactions and supporting self-efficacy expectations about the ability to effectively cope with adverse symptoms, particularly anxiety. Key instructional points of the OLP rationale protocol include awareness that placebo effects are powerful and have been shown to have effects that can be similar to those of analgesics (**Supplementary Table 1**). We also explain that doubts are not a problem as OLPs can be effective through conditioning, context and observation. Furthermore, we point out that patients compliance is critical and that placebos can unlock the body´s natural healing power (45).

##### 2.4.1.1 Video instruction

In the video, a patient shares her positive experience of using OLP to effectively reduce anxiety before surgery. This observational learning intervention via video builds on evidence supporting the beneficial effects of digital tools in surgical settings (41). Based on our knowledge of the role of self-efficacy as a contributor to the effects of OLPs (e.g., 43), the content of the video will be designed to enhance expectations of self-efficacy for coping with anxiety. This will also allow us to elucidate whether self-efficacy can be enhanced by observing others, resulting in more positive treatment expectations and perioperative health outcomes.

The patient in the four-minute video is played by a middle-aged woman matching the patient population (40–50 years old) sitting on a chair in a patient room. The patient talks about her experience and silent video sequences are shown of the anesthesia consultation, taking the placebo, the surgery preparation and her stay on the ward. The viewer sees how the patient takes the OLP pill and then relaxes. In the video, the patient again mentions the rationale of the OLP. She also explains her belief that she can influence her own situation with the OLP, reflecting self-efficacy as a mechanism underlying OLP effects.

#### 2.4.2 Blinding

As expectation modulation is central to the study, blinding will be ensured as follows: randomization will be performed prior to informed consent by a study researcher who is not involved in the initial screening and enrollment. The random allocation sequence and concealment is ensured by sealed envelopes. Group allocation with respect to OLP administration will only be communicated to the study physicians who will explain the study objectives and obtain patient’s informed consent based on version A or B of the consent form. Therefore, as the study physicians are necessarily unblinded with respect to OLP administration, they are also blinded with regard to video observation. The study staff showing the video to the OLP+V-Group is not blinded in this respect, but otherwise have no contact with the patients and are not involved in their care. The nursing staff on the ward who administers the OLP is also not blinded to the administration of the OLP. They are only blinded regarding the video. The anesthesiologists and gynecologists involved in the regular perioperative patient care of all study patients are completely blinded to the groups. In addition, we use several strategies to ensure that patients do not exchange information with each other. Based on consultation with gynecology colleagues, we assume that only a limited number of patients per day at each center will meet the inclusion and exclusion criteria. In addition, we coordinate the distribution of patients on the wards with case management, and the overall short length of stay of women in the clinic makes communication between patients unlikely.

Entering the data into a database, pre-processing of the data and the initial quality control and descriptive analyzes are carried out prior to unblinding.

### 2.5 Measures

#### 2.5.1 Psychosocial characteristics

Prior to experimental procedures, participants complete a psychosocial questionnaire battery. For characterization of participants, we will focus primarily on sociodemographics, anxiety as a state and trait (short-form State-Trait Anxiety Inventory (STAI; 46, 47) and the general health status using the Pain and State of Health Inventory (SBI; 48).

#### 2.5.2 Anxiety

Given the key role of anxiety in the experimental design of the PATE Trial, self-reported anxiety is measured using two different but complementary instruments: Preoperative Anxiety with the Numeric Rating Scale (NRS 0–10; 0 – No anxiety; 10 – Highest anxiety imaginable) and the Amsterdam Preoperative Anxiety and Information Scale (APAIS; 49, 50). This questionnaire consists of six items rated on a five-point Likert scale from “not at all” (1) to “extremely” (5). It represents the factors of anxiety and need for information regarding anesthesia and surgery. As a good indicator of its validity, this widely used questionnaire correlates with the State Anxiety Scale (STAI) with r=0.74, r=0.67 and r=0.64 and its retest reliability based on 42 subjects is r=0.92 for the anxiety scale and r=0.62 for the need for information scale (both p<0.001) (50). The brevity and clarity of this questionnaire made it particularly suitable for use in clinical settings where more complex questionnaires may not be acceptable to patients.

#### 2.5.3 Expectations

In order to examine the overall treatment expectations and their interactions with the OLP, the expectations in relation to the anesthesia and operation are recorded using the Stanford Expectation of Treatment Scale (SETS; 51) and in relation to the OLP using the Generic rating scale for previous treatment experiences, treatment expectations (GEEE; 52). The SETS consists of items that specifically measure patients’ degree of optimism or pessimism about the expected effects of their treatment. These items are structured to capture a range of expectations, from certainty of benefit to concern about possible ineffectiveness, each rated on a Likert scale, allowing researchers to systematically assess the impact of expectations on clinical outcomes before surgery (53). The GEEE assesses patients’ expectations of their specific treatment, in this case OLP. The scale includes measures of positive and negative expectations, previous experience with similar treatments, and the current effects of the treatment under investigation. It uses NRS to assess expectations of improvement and possible side effects. This comprehensive but brief approach provides a nuanced understanding of how patients’ expectations and previous experiences may influence their treatment outcomes in clinical settings (54, 55). Expectations are measured repeatedly to capture the impact of the study physician’s suggestions regarding the OLP and of the video.

#### 2.5.4 Postoperative pain

Postoperatively at T4 and T5, patients indicate their pain at rest and during movement (NRS 0–10; 0 – No pain at all; 10 – Highest pain imaginable). In addition, based on previous studies (23, 56), patients rate their pain based on localization, which includes “deep abdominal pain,” which is described as dull, cramping, and difficult to localize, and is indicative of visceral pain, usually from internal organs. In contrast, “superficial wound/scar pain” is described as somatic pain, which is typically more localized and associated with the skin, muscle or connective tissue around the surgical wound or suture (both assessed on Visual Analogue Scales (VAS 0–10; 0 – No pain; 10 – Highest pain imaginable). Furthermore, we record the subjective perception of pain relief and satisfaction with the pain therapy using the NRS.

#### 2.5.5 Patient perspective

In order to capture the patient’s perspective beyond their ratings of anxiety and pain, we examine attitudes towards the acceptability of OLPs as well as perceived characteristics of the doctors. For the latter, patients rate the perceived warmth and competence of the study physician in the consultation and of the anesthesiologists performing anaesthesia postoperatively, using 6 items each for warmth and competence (57). Each item is rated on a 5-point scale from 1 (not at all) to 5 (extremely).

To deepen our understanding of the patient perspective on the use of OLPs in the future, patients are asked postoperatively about the appropriateness and potential use of placebos. For this purpose, patients rate the appropriateness of taking a placebo before surgery on an NRS (NRS 0–10; 0 – Not appropriate; 10 – Very appropriate). We also measure self-efficacy, the extent to which an individual is confident that they have contributed to improve their preoperative well-being. Patients also give their opinion on whether they would consider taking a placebo in a similar or different situation in the future.

#### 2.5.6 Healthcare providers perspective

Furthermore, we are interested in the acceptability of OLPs by healthcare providers. The healthcare providers are asked about their view on anxiety reducing effects in the treated patient after the administration of the OLP assessed with an NRS (NRS 0–10; 0 – No effect; 10 – Very good effect).

#### 2.5.7 Clinical data

In order to analyze the effects of our intervention on objective measures such as medication requirements and medication intake, we record:

- previous illnesses, previous medication the preoperative consumption of sedatives
- medication administration and dosage (including hypnotics and analgesics) during induction of anesthesia
- medication, especially analgesics in the recovery room and in the post-operative phase

In addition, we record the type of surgery and times relevant in everyday clinical practice with regard to the duration of anesthesia induction, the operation and in the recovery room, as well as the length of hospital stay.

### 2.6 Data management

Both project coordinators (authors J.L.A. and J.W.) are part of the Collaborative Research Center 289 “Treatment Expectations”, which is responsible for conducting studies at both the Hamburg-Eppendorf and Essen sites. A standardized data protection concept was created for all projects of the Collaborative Research Center and the approval of the data protection officer of the University Medical Center Essen and the University Medical Center Hamburg-Eppendorf was obtained for further subprojects.

Apart from patient consent and the key pseudonym list, all data is exclusively pseudonymized, i.e. encrypted using a numerical code and stored accordingly. The participants are assigned a corresponding numerical code, which is used to identify questionnaires and electronic data.

The patients receive the questionnaire battery mainly in digital form on a tablet, but in paper form right before the induction of anesthesia and in the recovery room. To avoid losing data, we prefer the digital solution, but it’s not possible to use tablets immediately before surgery and in the recovery room, so patients are asked by staff and it’s documented on paper. For this, the open-source survey tool LimeSurvey (LimeSurvey GmbH, Hamburg, Germany) is administered by a central project of the CRC (58). Only the study investigators have access to the data (J.L.A. and J.W.). As the study is conducted collaboratively by two study physicians at only two already cooperating study centers within the CRC 298, no data monitoring committee is necessary.

### 2.7 Data analysis

A power analysis calculated with G*Power (version 3.1.9.6) resulted in a sample size of 120 patients for an expected small to medium effect size f of 0.2, two measurement time points, three groups, a significance level of α=0.05 and a power of 0.95. In order to be able to take possible exclusions during the course of the study into account, 20% more patients will be recruited, so that the final sample size is N=144 women. The effect size was derived from our own data and the literature after careful consideration with experts and colleagues at the two university hospitals. A medium to small effect size was chosen in order to be able to investigate small effects.

To analyze the main outcome (object 1), preoperative anxiety (APAIS), we will perform a univariate analysis of variance (ANOVA) with *post hoc* tests to determine differences in anxiety before surgery in the three groups. To analyze the dynamic changes of anxiety in the different groups before and after open placebo administration (TAU, OLP and OLP-V), repeated measures analyses of variance followed by *post hoc* tests are performed (object 2). In addition, ANOVAs will be conducted to assess postoperative pain (NRS) and other clinical characteristics by group (object 3). Patient and staff reports are primarily analyzed descriptively and free-text qualitatively (object 4). Furthermore, correlation and regression analysis will be used to identify exploratory predictive patient characteristics (object 3 & 4). No interim analysis is planned. If data is only available from one patient at three or fewer time points, the patients are excluded from the analysis.

## 3 Anticipated results

**Figure 2:**
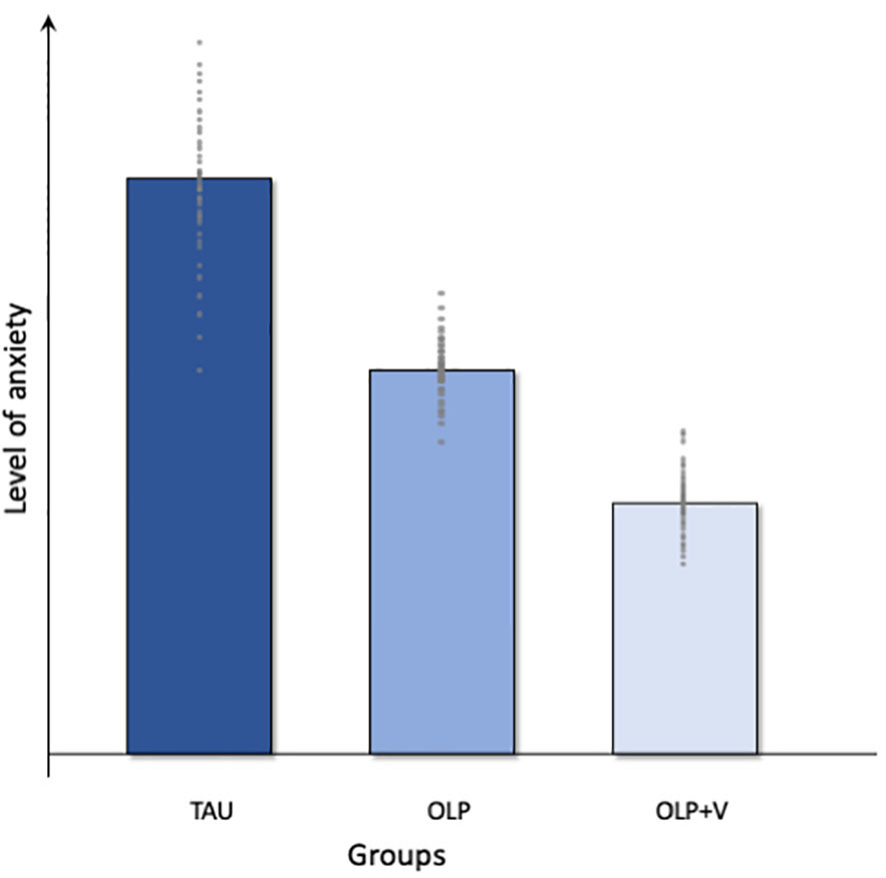
Graphical overview of the expected results. TAU, treatment as usual; OLP, open-label placebo; OLP+V, open-label placebo + video.

Given the background and the specific aims, we expect the following results based on the planned hypothesis testing of the data collected as part of the PATE Trial. A symbolic illustration of the expected group differences in the level of anxiety in the three study groups can be found in **Figure 2**.

Objective 1: To compare preoperative anxiety after standard treatment (TAU-Group) against modulation of treatment expectations using OLP (OLP-Group) and to determine whether the effects after modulation of treatment expectation using OLPs can be enhanced by a video with aspects of verbal suggestion and observational learning (OLP+V-Group).

We postulate a lower level of preoperative anxiety in the OLP groups compared to treatment as usual, e.g. the provision of information as part of the anesthesiological consultation, with the greatest effects in the OLP+V-Group (**Figure 2**). Therefore, we analyze the self-reported preoperative anxiety (APAIS), to determine differences in anxiety immediately before surgery across the three groups. Previous studies, such as those by Schaefer et al. (30, 59), and Buergler et al. (60), demonstrate the effectiveness of OLP in modulating negative emotions, such as anxiety and stress (30, 59, 60). In a study with test anxiety as the primary outcome, Schaefer et al. demonstrated how the use of OLPs could successfully contribute to its reduction in students (30). The same working group presented that self-reported lower emotional distress when viewing highly arousing negative pictures in the OLP-Group was associated with activation of brain regions known to modulate affective states, such as the hippocampus and periaqueductal grey (59). In turn, a positive correlation between anxiety and OLP response has already been shown, with gastrointestinal-specific anxiety beeing a predictor of response to OLPs in patients with irritable bowel syndrome (38). Therefore, we expect a reduced level of preoperative anxiety in the OLP groups. It should be noted, that the literature does not indicate the minimum clinically significant difference for the APAIS or anxiety on an NRS, therefore it should be considered that a statistically significant result does not necessarily imply clinical significance.

We hypothesize that the effect is pronounced in the OLP+V-Group, as the video with aspects of verbal suggestion and observational learning is particularly effective in reducing anxiety. Verbal suggestion and observational learning are important psychological mechanisms underlying positive and negative expectancy effects with great potential for clinical applications. Building on our previous work with expectation manipulation in chronic low back pain, e.g. observing a sham actor patient mimicking the benefits of pharmacological treatment (in this case amitriptyline) on function (42), we aim to support these findings in patients undergoing surgery. As there has been no study to date of OLP and observational learning in patients undergoing surgery, we aim to address this gap, given the potential benefits of using it in a video format in clinical practice.

Objective 2: To compare dynamic changes in anxiety and treatment expectations in the three groups.

In the context of the modulation of treatment expectations, we assume that not only the administration of the OLP alone, but rather the entire psychosocial treatment context, in particular the doctor–patient interaction, is pivotal. Therefore, we are interested in the dynamic changes in both preoperative anxiety and treatment expectations over time. Hence, we will analyze the course of self-reported anxiety based on the anxiety ratings (NRS) and treatment expectations (SETS) for the four time points, before and after OLP information, on the ward and directly before induction of anesthesia. We expect that the detailed consultation with the study physicians and the explanation of the rationale of the OLP will reduce patients’ preoperative anxiety and positively modulate their expectation.

Objective 3: Comparison of group differences in the pre- and postoperative course, in particular visceral and somatic postoperative pain intensity, sedative, anesthesia and analgesic requirements, duration of anesthesia induction and hospital stay.

Preoperative anxiety is associated with the risk of intraoperative complications and the need for sedation (5). It also impacts on postoperative outcomes (6, 7, 61), especially the pain experience (8, 9).

In addition to reduced anxiety, we expect that patients in the OLP-Group will require less benzodiazepine rescue medication. In the postoperative period, we expect patients in the OLP groups to report less pain than those in the TAU-Group. A difference of at least 1 point on the NRS is considered a clinically relevant difference, as the MCID for acute pain management is based on a systematic review of at least 8 mm on a VAS scale (62). Our assumption is based on the close relationship between anxiety and pain (8, 9). In particular, visceral pain is associated with anxiety and negative emotions (63), leading us to hypothesize that visceral pain will be particularly reduced with OLP. Exploratively, we will examine analgesic consumption in the PACU and postoperatively on the ward, as reduced preoperative anxiety may be associated with reduced postoperative opioid consumption. We also aim to assess whether there are group differences in patients’ reported self-efficacy and the appropriateness of placebo use in the clinical context from the patient’s perspective.

Objective 4: To explore the patient and healthcare providers perspective regarding the use of OLPs to reduce anxiety before surgery.

Research on open-label placebo in the clinical context remains novel, meaning there is little data on the patient and healthcare provider perspective. To date, quantitative research in clinical populations is lacking and the healthcare provider perspective, e.g. those who administer OLPs, has not yet been captured (43, 44, 64).

Therefore, one aim of this study is to explore patients’ views on the effectiveness and appropriateness of OLPs before surgery in a large clinical population. Firstly, we investigate the effectiveness of OLPs from the patient’s perspective post-surgery. We further intend to gain an understanding of whether patients consider OLP administration to be critical in the context of surgery or are open to it, thus asking their opinion on the application in this and other situations. In addition, we plan to interview the TAU-Group who have not received OLPs for their views on the application of OLPs. In addition to the patient perspective, we include the health care providers opinion on patients’ benefit from taking OLPs.

## 4 Discussion

### 4.1 Advantages, limitations, pitfalls

The PATE Trial has both strengths and limitations. From a clinical perspective, the main strength of this trial is the potentially large patient population that could benefit from the reduction of preoperative anxiety with OLP. Every day, hundreds of thousands of operations are performed worldwide (4), and many patients are affected by preoperative anxiety, resulting in increased morbidity (61). Current treatment regimens are inadequate and there is an urgent need for low side effect and clinically feasible alternatives. The potential impact of this study, conducted in a selected at-risk population, is considerable and potentially powerful.

Another strength of this study relates to the use of OLPs in a promising new area of preoperative anxiolysis that has not yet been explored. The indication for OLP administration with a positive rationale provides patients with a side-effect free option to reduce their preoperative anxiety in addition to the usual standard treatment. The use of OLP does not expose patients to any additional risk and they will not be deprived of therapy if they require it. In contrast, a possible reduction in the use of anxiolytic drugs may protect patients from unwanted side effects and drug interactions. Previous studies with OLP have shown promising results in the past, especially for subjective complaints such as anxiety as described above (30, 59, 60). However, the conditions for which the use of OLPs is beneficial are still under discussion (65). In the context of anxiety before surgery and anxiety before anesthesia, we consider the use of OLPs, potentially contributing to patient empowerment, to be a suitable indication and consequently designed this study.

In addition, we are taking a patient-centred approach and investigate their perspectives on the use of OLPs. We believe that this information has the potential to be of great value in the discussion of clinical application, given the limited experience with OLPs in the clinical settings to date. Observational learning strategies have also rarely been used systematically in clinical settings. Almost all of the existing data are from human experimental studies (33). Notably, implementation via video is easy to realize in clinical practice and may yield additional synergistic benefits. These hypothesis-generating approaches within clinical trials provide valuable translation input to extend research on placebo effects in general.

Another strength of the study design is the bicentric recruitment with highly standardized procedures at both clinic sites, enhancing generalizability of results. Furthermore, we include a true treatment-as-usual control group, which receives the same medical treatment and is drawn from the same patient population as the intervention groups.

The knowledge gained from this study will pave the way for future clinical research to test whether and how these results generalize to other patient populations and treatment contexts. They promise to optimize the efficacy, tolerability, safety and cost-effectiveness of preoperative anxiety management strategies. Ultimately, the knowledge gained may provide a basis for extrapolating these approaches to treatment strategies to prevent or improve anxiety and pain in a wide range of patients undergoing surgical procedures. This project will expand knowledge on how treatment expectations and their mechanisms can be systematically enhanced in the clinical context to improve treatment outcomes.

Limitations and potential pitfalls are equally important to consider.

A well-known problem in OLP trials is the need for unblinding, which is not an easy obstacle to overcome in OLP trials that require communication of the rationale. However, all other parts of the trial are single-blinded and all healthcare professionals performing the standard medical procedures are double-blinded. Although we have developed a guideline for communicating the rationale for OLP, we cannot rule out the possibility of slight variations in patient conversation resulting in differences in the effect of OLP administration. In addition, confounding cannot be excluded due to the bicentric design with different procedures and different gender of the study physicians at the two study centers, although the bicentric design is also a strength. To minimize possible differences, all procedures are standardized and controlled to the greatest possible extent. Because we cannot rule out the possibility that the study physician’s attitude towards open-label placebos may have an influence on the outcome, we minimize the time spent by the study physician and the patient in consultation only to the study enrollment and OLP instructions, and secondly we capture the patients’ perspective on the perceived characteristics of the physicians (see 2.5.5.). Furthermore, the power analysis was not designed for conducting further subgroup analyses and possibly smaller effects cannot be recorded. The calculation of an ANOVA does not take random effects into account, so it may make sense to use a different statistic approach. The postoperative situation in particular may also depend on the clinical outcome of the surgery, resulting in smaller effects not being detectable.

Furthermore, it cannot be excluded that simply showing a video after explaining the study and the rationale has an anxiolytic effect, regardless of the content. However, it proved difficult to find a truly neutral video to show before surgery, as patients are particularly vulnerable at this time. The concept of a “neutral” video in this context is problematic; almost any content could be unintentionally distracting or, more worryingly, potentially induce nocebo effects. After careful consideration, we decided not to include such a video in our trial. To minimize any “dose” effect of the additional video exposure, the study physician leaves the room after the OLP briefing and a trained assistant then starts the video. The findings of studies on the influence of a video as part of information on the perioperative anxiety experience of patients are heterogeneous, but a video does not appear to contribute per se to a reduction in anxiety in patients compared to patients without watching a video (66). In addition, there is so far little knowledge and data regarding the content of a treatment expectation optimizing and anxiety reducing video. Consequently, a possible influence of verbal suggestion and observational learning on the effect of OLPs needs to be carefully interpreted.

Our study population consists exclusively of female participants, who have a higher prevalence of preoperative anxiety, but as a result we can only make limited general statements about preoperative anxiety. Another limitation is that our treatment-as-usual (TAU) group as control does not allow us to make a clear statement about the effectiveness of any prescribed anxiolytic medication and compare it with the effect of OLP.

## 5 Conclusions

One of the most common concerns of patients undergoing surgery is preoperative anxiety, accompanied by increased risk of postoperative pain and worse outcomes. Current treatment of preoperative anxiety is often limited as sedatives cause side effects and their effectiveness is still debated. Placebo research has shown that optimizing positive treatment expectations, which can be achieved through the administration of placebos and information, has clinically relevant effects on anxiety, pain and treatment outcomes in studies. This bicentric, prospective, randomized-controlled clinical trial “PATE” aims to alleviate preoperative anxiety by optimizing positive treatment expectations facilitated by OLP and potential reinforcement of these effects through observational learning via a positive expectation-reinforcing video. To achieve these objectives, patients undergoing gynecologic laparoscopic surgery receiving an OLP with a positive rationale and a group additionally viewing a video presenting information from a satisfied patient will be compared to standard care. The impact on preoperative anxiety and postoperative experience, particularly visceral and somatic postoperative pain report outcomes will be analyzed. As the non-deceptive administration of placebos can lead to positive outcomes without side effects when indicated and current treatment of preoperative anxiety is limited, the findings from clinical placebo research have the potential to improve outcomes and patient experience in the surgical setting.

## Ethics statement

Ethical approval for this study was obtained in coordinated process in February 2024 of the ethics committees at University Hospital Essen (23–11529-KOBO) and Hamburg (2023–200843-BO-kV-bet). The study has been registered in the German Registry for Clinical Studies (DRKS) and is publicly available there (DRKS00033221). The findings of the study will be published via open access in peer-reviewed journals and disseminated through presentations at national and international conferences. Additionally, the data will be delivered to the public and interested participants in cooperation with our science communication team of the CRC 289. The studies were conducted in accordance with the local legislation and institutional requirements. The participants provided their written informed consent to participate in this study.

## Author contributions

JW: Conceptualization, Data curation, Formal analysis, Funding acquisition, Investigation, Methodology, Project administration, Resources, Software, Supervision, Validation, Visualization, Writing – original draft, Writing – review & editing. RK: Conceptualization, Funding acquisition, Methodology, Supervision, Writing – review & editing. SB: Conceptualization, Funding acquisition, Methodology, Supervision, Writing – review & editing. TB: Conceptualization, Methodology, Supervision, Writing – review & editing. SE: Conceptualization, Funding acquisition, Methodology, Supervision, Writing – review & editing. JA: Conceptualization, Data curation, Formal analysis, Funding acquisition, Investigation, Methodology, Project administration, Resources, Software, Supervision, Validation, Visualization, Writing – original draft, Writing – review & editing.
