# Corrigendum: Preoperative Anxiolysis and Treatment Expectation (PATE Trial): open-label placebo treatment to reduce preoperative anxiety in female patients undergoing gynecological laparoscopic surgery – study protocol for a bicentric, prospective, randomized-controlled trial

**DOI:** 10.3389/fpsyt.2024.1497098

**Published:** 2024-10-15

**Authors:** Johannes Wessels, Regine Klinger, Sven Benson, Thorsten Brenner, Sigrid Elsenbruch, Jana L. Aulenkamp

**Affiliations:** ^1^ Department of Anesthesiology, University Medical Center Hamburg Eppendorf, Hamburg, Germany; ^2^ Institute for Medical Education, Center for Translational Neuro- and Behavioral Sciences (C-TNBS), University Hospital Essen, University of Duisburg-Essen, Essen, Germany; ^3^ Department of Anesthesiology and Intensive Care Medicine, University Hospital Essen, University Duisburg-Essen, Essen, Germany; ^4^ Department of Neurology, Center for Translational Neuro- and Behavioral Sciences (C-TNBS), University Hospital Essen, University Duisburg-Essen, Essen, Germany; ^5^ Department of Medical Psychology and Medical Sociology, Ruhr University Bochum, Bochum, Germany

**Keywords:** placebo, treatment expectation, postoperative pain, anxiolysis, visceral pain, somatic pain, fear, surgery

In the published article, there was an error in the author list, which erroneously included Christian Zöllner as an author. The corrected author list appears below:

“Johannes Wessels^1^, Regine Klinger^1^, Sven Benson^2^, Thorsten Brenner^3^, Sigrid Elsenbruch^4,5^ and Jana L. Aulenkamp^3,4^*”

The authors apologize for this error and state that this does not change the scientific conclusions of the article in any way. The original article has been updated.

