# Supplementary material for: Preoperative Anxiolysis and Treatment Expectation (PATE Trial): open-label placebo treatment to reduce preoperative anxiety in female patients undergoing gynecological laparoscopic surgery – study protocol for a bicentric, prospective, randomized-controlled trial
Source: Front Psychiatry. 2024 Jul 9;15:1396562. doi: 10.3389/fpsyt.2024.1396562 (PMC11265268; doi:10.3389/fpsyt.2024.1396562)
Supplement: Supplementary file 1 [file Table_1.docx]

Supplement 1: **Wording of the rationale for the open-label placebo provided by the study physician, translation from German**

- The novelty of the study is that we offer you placebos before the operation and of course tell you that they are placebos. We will now explain a lot about the effect of the placebo. In the end, your body will handle the anxiety on its own. You take a placebo and your brain manages to keep them under control. Placebos are pharmacologically non-active substances, but not ineffective ones!
- The placebo effect has been known for many years. This effect activates the body's own "pharmacy". During this process, messenger substances, e.g. neurotransmitters, are released. Amazingly, these have the same effect on many patients as medication!
- (Just in case of doubt: nature and evolution have developed this process so that the body can also regulate pain or anxiety itself. Sometimes this is very important, for example when fear or pain would prevent you from getting to safety in a dangerous situation).
- **How does this powerful placebo effect work?**
- Our body learns from experiences that we store in our brain over the years: We are given pills as children and learn that "this pill helps me with my pain" or "this pill helps me to calm down". The body stores the "pill" symbol and associates it with the soothing effects you have learned!
- We also learn the placebo effect by observing others! If you see your neighbour in hospital being given a medicine that helps her to relax, then the same medicine, or a tablet that looks like it, may have a similar effect on you.
- The important thing is to take it consciously! Words such as "This tablet is helping me now!" are also like a ritual.
- The effectiveness of the placebo can be enhanced by positive expectations. Confidence in the treatment is very important, but not essential.
- Placebos can therefore have an anxiety-reducing and therefore pain-reducing effect through self-efficacy, i.e. through yourself and your body's own pharmacy.
- Keep an open mind and observe if the placebo is working for you.
